# Herbal Tea Essences (HTE) Ameliorate HFD-Induced Obesity

**DOI:** 10.1155/2022/9315318

**Published:** 2022-11-21

**Authors:** Yue Wang, Ying Han, Rongfu Lv, Chengyong He, Zhenghong Zuo, Ying Chen, Jiyi Huang

**Affiliations:** ^1^Clinical Research Center for Chronic Glomerular Disease, Department of Nephrology, The Fifth Hospital of Xiamen, Xiang'an Branch of the First Affiliated Hospital, The First Affiliated Hospital of Xiamen University, School of Medicine, School of Life Sciences, Faculty of Medicine and Life Sciences, Xiamen University, Xiamen 361102, China; ^2^The Department of Laboratory Medicine, The First Affiliated Hospital of Xiamen University, Xiamen 361101, Fujian, China; ^3^Xiamen Key Laboratory of Genetic Testing, Xiamen 361101, Fujian, China; ^4^Xiamen Herbt Biotechnology Company Limited, Xiamen, Fujian 361005, China

## Abstract

Tea is one of the most popular beverages in the world. The health-promoting effects of tea and its individual constituents, including antiobesity and antihyperlipidaemia effects, have been well accepted. In this study, we evaluated the effects of herbal tea essence (HTE), a commercial product extracted from black tea, on HFD-induced obesity in mice. HTE effectively reduces the gain in body weight and improves glucose tolerance and insulin sensitivity after HFD treatment. HTE inhibits lipid accumulation in the body and reduces serum lipid contents. Furthermore, HTE negatively regulates the expression levels of genes that control lipogenesis and gluconeogenesis and upregulates the expression of genes for lipid *β* oxidation. The regulatory effects of HTE on these genes may occur through activation of the AKT, IRS-1, and AMPK signalling pathways. Our observations suggest that HTE could be a promising option for nutritional intervention in the treatment of obesity.

## 1. Introduction

Obesity, a nutritional and metabolic disorder, has been recognized as a major risk factor for diabetes, cardiovascular diseases, and other kinds of diseases [1, 2]. Increasing consumption of Western diets is a major cause of obesity [3]. Currently, obesity is an epidemic in industrialized countries and significantly threatens public health [4]. Therefore, researchers have made great efforts to develop strategies to prevent obesity and related metabolic syndromes. Among those strategies, herbs have drawn great attention due to their promising antidiabetic and antiobesity effects [5–11].

Tea is one of the most popular beverages in the world [12]. As reported, tea has beneficial antioxidative [13], anti-inflammatory [14], antiarthritic [15], and antiangiogenic [16] effects. Currently, the protective effects of tea against obesity have become a popular topic in nutrient and food research. A variety of research groups have reported the antiobesity and hypolipidaemic effects of tea in animals and humans [17–19].

To date, the biological effects of tea and its individual constituents have been intensively investigated [20]. Polyphenols are enriched in both green tea (unfermented) and black tea (fully fermented) [21, 22]. Several studies have suggested that polyphenols exhibit antidiabetic and antiobesity effects [23–26]. Catechins, the main derivatives of polyphenols, form tea pigments after fermentation [27, 28]. Among the constituents of the tea pigment, theabrownin (TB) is the most important for the pharmacological effects of fermented tea [29]. As reported, TB showed anticarcinogenesis and antihyperlipidaemia effects [30]. However, studies that have focused on the effects of TB on obesity are still lacking.

As the ingredients of black tea can be greatly changed during different fermentation processes [31], TB content significantly varied among tea products. The proportion of TB determines the effect of black tea on health. Instable fermentation processes can largely alter the TB content and compromise the quality of the black tea. To solve this problem, we deeply processed black tea and developed a black tea product named herbal tea essences (HTE), which contained a stabilized and high proportion of TB and low proportions of polyphenols and caffeine.

In this study, we tested the effect of HTE on high-fat diet (HFD)-induced obesity. We found that HTE administration sufficiently and effectively reduced the weight gain caused by HFD feeding. Our findings suggest that HTE could be a promising option for the treatment of obesity.

## 2. Materials and Methods

### 2.1. Animals

Eight-week-old C57BL/6 male mice were purchased and maintained in the Xiamen University Laboratory Animal Center. All animal experiments were approved by and performed according to the experimental guidelines of the Animal Care and Use Committee of Xiamen University.

### 2.2. High-Fat Diet and HTE Treatment

Mice were fed a normal chow diet (NCD) or HFD (Research Diet, D12492). For HTE treatment, low (0.05 g/kg), middle (0.2 g/kg), or high (1 g/kg) doses of HTE were administered to the HFD-fed mice by gavage administration (i.g., once per day. After 6 weeks of HTE treatment, the mice were sacrificed and tissue samples were collected for the following experiments.

HTE is a black tea extract produced by Xiamen Herbt Biotechnology Company Limited. It contains 79% theabrownins, 8.46% polyphenols, 2.76% caffeine, and 9.8% water-soluble compounds.

### 2.3. Glucose Tolerance Test (GTT) and Insulin Tolerance Test (ITT)

The GTT and ITT tests were performed as previously described [32]. Briefly, in the GTT test, mice were fasted for 16 h and then received an intraperitoneal (ip) injection of D-glucose (2 g/kg, Sigma). For ITT, mice were fasted for 4 h and then received an intraperitoneal (ip) injection of insulin (0.5 units/kg). Blood glucose concentrations were measured at the indicated time points using a glucometer (Sinocare, Inc.).

### 2.4. Serum Component Analysis

Mouse serum samples were prepared as previously described [32]. Serum biochemical indices were tested with a Cobas 8000 c702 module analyser (Roche, Rotkreuz, Switzerland) using detection kits (Mindray, China) for the following molecules: alanine aminotransferase (ALT, 105-000442-00), aspartate aminotransferase (AST, 105-000443-00), triglyceride (TG, 105-000449-00), cholesterol (TC, 105-000448-00), and low-density lipoprotein cholesterol (LDL-C, 105-000464-00).

### 2.5. H&E Staining

Liver and epididymal adipose tissues were collected and fixed at the end of the experiment. After deparaffinization and rehydration, the sections were stained with haematoxylin solution (Sigma Cat# HHS16) for 8 min and rinsed for 3 min. Then, the sections were further stained with eosin solution (Sigma Cat# HT110316) for 2 min. After dehydration with graded alcohol and clearing in xylene, the mounted slides were photographed using an Olympus BX53 microscope [32].

### 2.6. Real-Time Quantitative PCR

Total mRNAs were extracted using TRIzol (Takara Cat# 9109) according to the manufacturer's instructions. cDNAs were prepared from 1 *μ*g total RNA using HifairTM II 1st Strand cDNA Synthesis SuperMix for qPCR (Yeasen Cat# 11123ES60) according to the manufacturer's instructions. Quantitative PCRs were performed using HieffTM qPCR SYBR Green Master Mix (Yeasen Cat# 11201ES08). The primer sequences are shown in the Supplementary table 1.

### 2.7. Western Blot Analysis

The liver samples were lysed in RIPA buffer (50 mM Tris-HCl, pH 7.5, 150 mM NaCl, 1 mM EDTA, 1% Triton X-100, 1% deoxycholate, 0.1% SDS) supplemented with protease inhibitor cocktail (MCE Cat# HY-K0010), phosphatase inhibitor cocktail I (MCE Cat# HY-K0021), and phosphatase inhibitor cocktail II (MCE Cat# HY-K0022) on ice for 30 min. The lysate was then centrifuged at 12,000 rpm for 30 min. Protein concentrations were measured using the BCA Protein Assay Kit (Sangon Biotech Cat# C503021). Proteins were resolved by 12% SDS‒PAGE and transferred to PVDF membranes (GE Healthcare Cat# 10600023). The membranes were blocked with 3% BSA in TBS (50 mM Tris-HCl, pH 7.5, 150 mM NaCl) for 1 h and then incubated overnight at 4°C with primary antibodies. After 3 washes with TBS plus 0.05% Tween 20, the membranes were incubated with secondary antibodies for 1 h and developed using an ECL kit (Millipore Cat# WBKLS0500). The following primary antibodies were used: p-AKT (CST, #4060), AKT (CST, #4685), p-IRS-1 (CST, #2385), IRS-1 (CST, #2382), p-AMPK (CST, #4184), AMPK (CST, #2532), and GAPDH (CST, #5174).

### 2.8. Statistical Analysis

The sample sizes (*n*) are indicated in the figure legends. All statistical analyses were performed using GraphPad Prism version 8.0. The data were presented as the mean ± SEM. A value of *P* < 0.05 was regarded as statistically significant.

## 3. Results

### 3.1. HTE Ameliorates High-FatDiet-Induced Obesity

**Figure 1:**
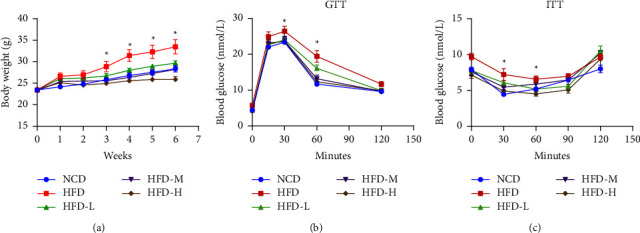
HTE inhibits HFD induced obesity and ameliorates metabolic disorders. Mice were fed NCD/HFD, and they received vehicle or low (HFD-L, 0.04 g/kg), medium dose (HFD-M, 0.2 g/kg), or high dose (HFD-H, 1 g/kg) HTE treatment for 6 weeks. Body weights were measured weekly (a). GTT was performed after 5 weeks of HTE treatment, and ITT was performed after 6 weeks of HTE treatment. (*n* = 6–8 mice per group). Each value represents the mean ± SEM. ^*∗*^, *P* < 0.05; one way ANOVA test.

We first used an HFD-induced obesity model to test the effects of HTE on obesity. Mice were fed a normal chow diet (NCD) or a high-fat diet (HFD) with 60% calories from fat. All mice were pretreated with HFD for 1 week and then administered different doses of HTE for another 6 weeks before sacrifice. During HFD feeding, HFD-fed mice without HTE treatment gained body weight rapidly. HTE treatment dose dependently and significantly inhibited the body weight increase induced by HFD feeding, and 1 week of high-dose HTE treatment sufficiently reduced body weight. Low and middle doses of HTE reduced body weight gain after 3 weeks of treatment. After 6 weeks of low- and middle-dose HTE treatment, the body weight of HFD-fed mice returned to a level comparable to that of NCD-treated mice (Figure 1(a)). In particular, high doses of HTE treatment even made HFD-fed mice thinner than NCD-fed mice, while no significant liver injury was observed, as indicated by serum AST and ALT levels at the end of the experiment (Figure S1). In addition to the ameliorative effect on obesity, HTE also reduced metabolic disorders, as indicated by the results of the glucose tolerance test (GTT) and insulin tolerance test (ITT) (Figures 1(b) and 1(c)). Low and middle doses of HTE significantly improved glucose clearance and increased insulin sensitivity. All doses of HTE treatment had no impact on daily food consumption in these mice (Figure S2).

### 3.2. HTE Promotes Lipid Metabolism

**Figure 2:**
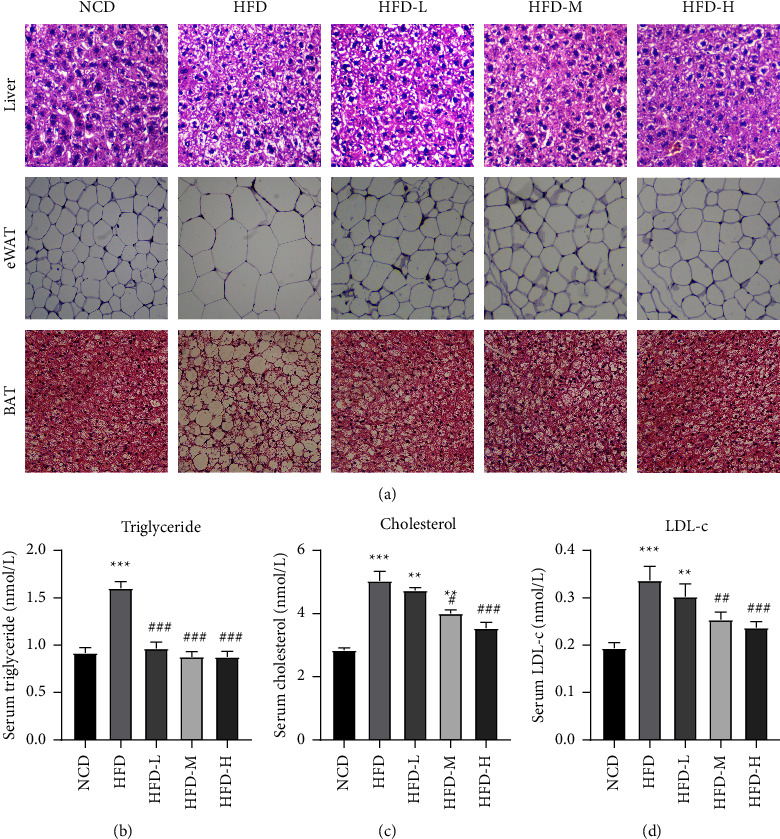
Treatment with HTE improves lipid metabolism. Mouse liver, epididymal adipose tissues (eWAT) and serum samples were collected at the end of the experiment as described in the materials and methods section. (a) Representative images of H&E stained liver, eWAT, and BAT. Scale bar, 250 *μ*m. (*n* = 6–8 mice per group). Each value represents the mean ± SEM. ^*∗∗*^, *P* < 0.01; ^*∗∗∗*^, *P* < 0.001, compared to the NCD group; ^#^, *P* < 0.05; ^##^, *P* < 0.01; ^###^, *p* < 0.001, compared to the HFD group, one way ANOVA.

In addition to improving metabolic disorders, HTE treatment inhibited HFD-induced hepatic steatosis. According to the H&E staining analysis, the HFD-fed mice had severe lipid accumulation in the liver. After HTE treatment, the accumulated lipids were greatly reduced (Figure 2(a)). Similar effects were observed in white adipose tissue (WAT) and brown adipose tissue (BAT). Treatment with HTE reduced the content of epididymal fat and the sizes of adipocytes (Figures 2(a) and S3). Furthermore, when we examined the serum lipid contents, HTE treatment reduced the serum cholesterol, triglyceride, and LDL-C contents (Figures 2(b)–2(d)). In summary, HTE protected mice from lipid accumulation after HFD feeding.

### 3.3. HTE Modulates the Expression of Genes That Control Lipid and Glucose Metabolism

**Figure 3:**
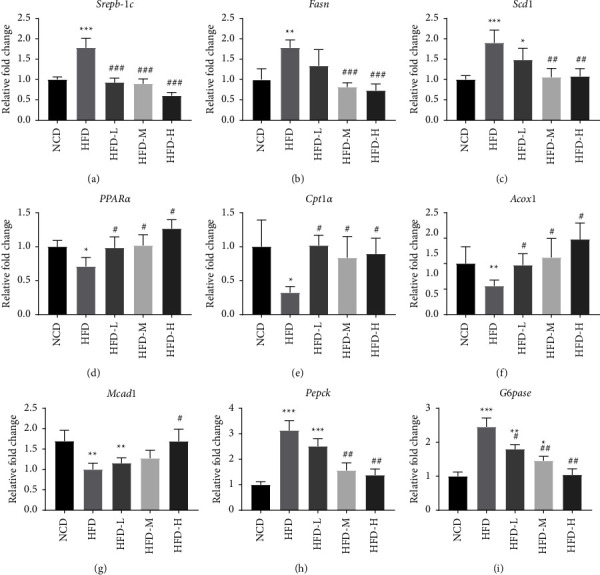
HTE treatment regulates the expression of genes that metabolize lipids and glucose. Total mRNA samples were collected from the liver at the end of the experiment. Real time PCR was performed to analyse the expression of the genes as indicated (*n* = 6 mice per group). Each value represents the mean ± SEM. ^*∗*^, *P* < 0.05; ^*∗∗*^, *P* < 0.01; ^*∗∗∗*^, *P* < 0.001 compared to the NCD group; ^#^, *P* < 0.05; ^##^, *P* < 0.01; ^###^, *P* < 0.001, compared to the HFD group, one-way ANOVA.

To explore the reason for the reduced lipid accumulation with the treatment, we examined the expression levels of the genes involved in lipid metabolism in the liver. As shown, after HTE treatment, the expression levels of lipogenesis genes, including Srebp-1c, Scd-1, and Fasn, were significantly reduced (Figures 3(a)–3(c)). At the same time, the expression levels of the proteins catalysing lipid *β*-oxidation, such as Ppar*α*, Cpt-1*α*, Acox1, and Mcad1, were greatly increased (Figures 3(d)–3(g)). Therefore, HTE inhibits lipid accumulation by suppressing lipid biosynthesis and increasing lipid clearance.

Meanwhile, we examined the expression of the enzymes catalysing gluconeogenesis in the liver. Pepck and G6pase expression levels were induced upon HFD feeding. In the mice receiving HTE treatment, the expression levels of these two genes were largely reduced, suggesting that HTE suppressed gluconeogenesis upon HFD feeding, resulting in improved glucose tolerance and insulin sensitivity (Figures 3(h) and 3(i)).

### 3.4. HTE Treatment Activates AKT, AMPK, and Insulin Signalling in the Liver

**Figure 4:**
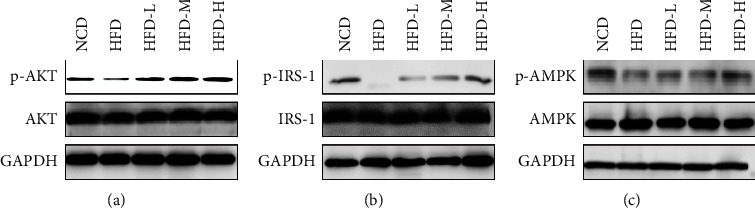
HTE activates signalling pathways controlling lipid and glucose metabolism. Protein samples were collected from the liver tissues at the end of the experiment as described. Western blot analyses were performed to measure protein levels using the indicated antibodies (*n* = 3 mice per group).

We then attempted to determine the molecular basis of the effects of HTE on glucose and lipid metabolism. We assayed the activities of the signalling pathways involved in lipid and glucose metabolism. As shown, HTE treatment significantly increased the levels of p-AKT, p-IRS-1, and p-AMPK in the liver tissues of HFD-fed mice, suggesting its activating effects on these key pathways that control glucose and lipid metabolism (Figures 4(a)–4(c)). Thus, HTE inhibits gluconeogenesis and lipogenesis and promotes lipid clearance, leading to the alleviation of metabolic disorders by HFD-treatment.

## 4. Discussion

In this study, we evaluated the effects of HTE on HFD-induced obesity in mice. Mice with daily HTE administration maintained their lean phenotype after 8 weeks of HFD feeding. They also showed normal glucose tolerance and insulin sensitivity. Treatment with HTE led to hypolipidaemia, as it suppressed lipid accumulation in the liver and adipose tissues and reduced serum lipid contents. On a molecular basis, HTE treatment suppressed the expression of the enzymes that catalyse lipogenesis and gluconeogenesis and promoted the expression of lipid *β*-oxidation genes in the liver. The modulating effects of HTE on these genes came from the activation of the insulin, AMPK, and AKT signalling pathways. Our findings therefore identify a new tea product that is beneficial for the control of obesity.

Tea is one of the most popular drinks in the world. Among the ingredients in tea, the antidiabetic and antiobesity effects of polyphenols have been widely studied [23–26]. However, the effects of TB, the major ingredient in black tea, on obesity and diabetes remain poorly studied. In our current study, we focused on the effects of HTE, a black tea product containing a high TB content, on obesity and its correlated metabolic syndromes.

We evaluated the effects of HTE on lipid and glucose metabolism in the HFD-induced obesity mouse model. We administered the mouse HTE treatment by i.g., daily, with a comparable dosage (the middle HTE dose) to that suggested for daily consumption in humans. Meanwhile, we also set up groups with low or high HTE dosages to better show the effects of HTE. As shown in the results, HTE treatment had significant dose-dependent effects on body weight gain, blood glucose level, serum lipid level, and lipid accumulation in tissues after HFD treatment. In particular, although the high dose of HTE had more significant effects, the mice receiving the middle dose of HTE largely recovered, showing results comparable to those of NCD treated mice.

For mechanistic studies, we examined the effects of HTE on signalling pathways and the expression of genes that control glucose and lipid metabolism in the liver. We found that insulin, AKT, and AMPK signalling were affected by HTE, resulting in modulation of the expression levels of lipid and glucose metabolism-related genes. It has been reported that tea or tea products can prevent HFD-induced obesity by several mechanisms, such as activating fat browning [33, 34], modulating gut microbiota [35, 36], and influencing lipid metabolism [37]. Our study showed that HTE had significant effects on lipid metabolism. We also believe that there are other effects of HTE that remain to be discovered.

Controlling obesity is a very complicated process. It could be a synergistic effect of various regulatory mechanisms in tissues and systems, such as the endocrine system, digestive system, immune system, and nervous system [38]. During this process, multiple signalling pathways are involved. Here, we mainly studied the effects of HTE on obesity in liver and adipose tissue and found that AKT, AMPK, and insulin signalling played roles in those tissues. We believe that there must be other signalling pathways affected by HTE. We will examine the effects of HTE on other systems, especially the nervous system, in the future.

## 5. Conclusion

In summary, our current study showed the effects of HTE on the improvement of obesity. HTE improved glucose tolerance and insulin sensitivity and suppressed lipid accumulation on liver and adipose tissues. Mechanistically, HTE improved lipid and glucose metabolism by activating key signalling pathways and modulating the expression of key genes involved in these two processes. As HTE administration was helpful to improve glucose and lipid metabolism, and HTE consumption has been shown to be safe [39], HTE should be expected to have marketing prospects.

## Data Availability

The data that support the findings of this study are available from the corresponding author upon reasonable request.

## Supplementary Materials

Supplementary Materials Supplementary Figure 1. Treatment with HTE does not trigger liver damage. Serum samples were collected as mentioned in Figure 2. AST and ALT levels were measured as indicated in the Materials and Methods section (*n* = 6 mice per group). Each value represents the mean ± SEM. Supplementary Figure 2. Treatment with HTE does not change food intake. The food consumption of the mice was monitored after 5 weeks of HFD treatment (*n* = 6 mice per group). Each value represents the mean ± SEM. Supplementary Figure 3. HTE treatment reduces the content of eWAT upon HFD treatment. The eWAT samples were collected and weighed at the end of treatment (*n* = 6 mice per group). Each value represents the mean ± SEM. ^*∗∗*^, *P* < 0.01; ^*∗∗∗*^, *P* < 0.001; one-way ANOVA test. Supplementary table 1. The primer sequences used for qPCR analysis.
