# Supplementary material for: Herbal Tea Essences (HTE) Ameliorate HFD-Induced Obesity
Source: Evid Based Complement Alternat Med. 2022 Nov 21;2022:9315318. doi: 10.1155/2022/9315318 (PMC11401730; doi:10.1155/2022/9315318)
Supplement: Supplementary Materials — Supplementary Figure 1. Treatment with HTE does not trigger liver damage. Serum samples were collected as mentioned in Figure 2. AST and ALT levels were measured as indicated in the Materials and Methods section (n = 6 mice per group). Each value represents the mean ± SEM. Supplementary Figure 2. Treatment with HTE does not change food intake. The food consumption of the mice was monitored after 5 weeks of HFD treatment (n = 6 mice per group). Each value represents the mean ± SEM. Supplementary Figure 3. HTE treatment reduces the content of eWAT upon HFD treatment. The eWAT samples were collected and weighed at the end of treatment (n = 6 mice per group). Each value represents the mean ± SEM. ∗∗, P < 0.01; ∗∗∗, P < 0.001; one-way ANOVA test. Supplementary table 1. The primer sequences used for qPCR analysis. [file 9315318.f1.docx]

**Supplementary figure 1**

**
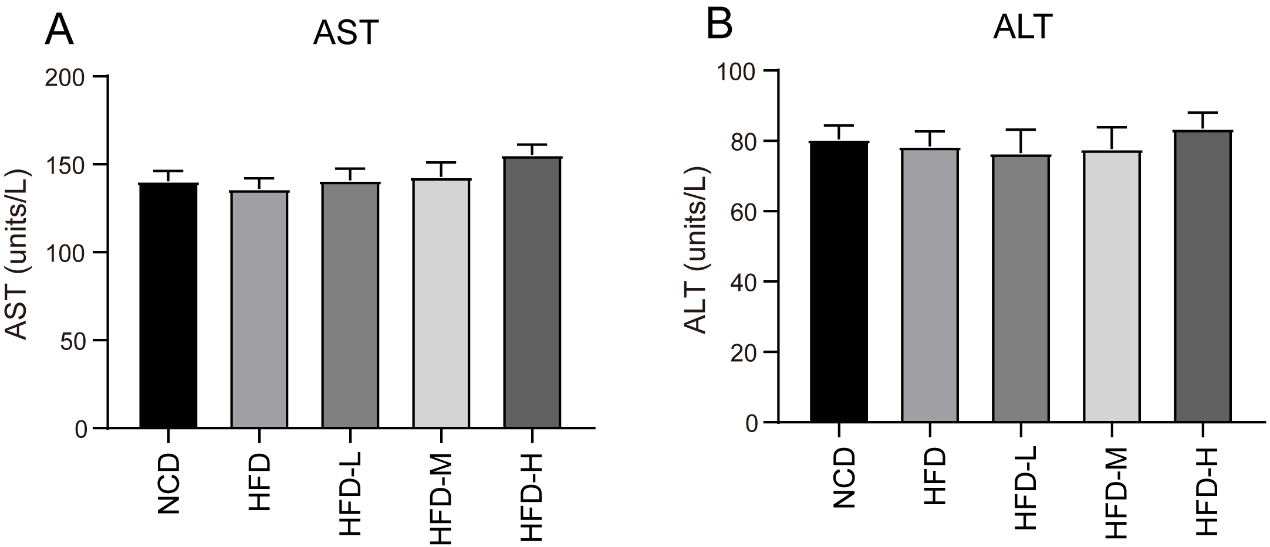
**

**Supplementary figure 2**

**
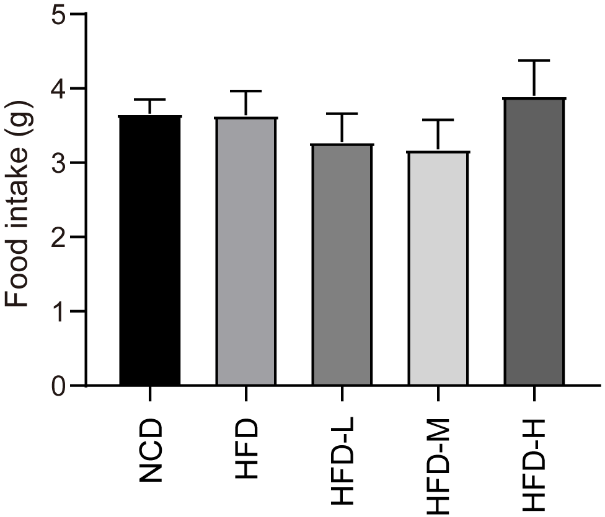
**

**Supplementary figure 3**

**
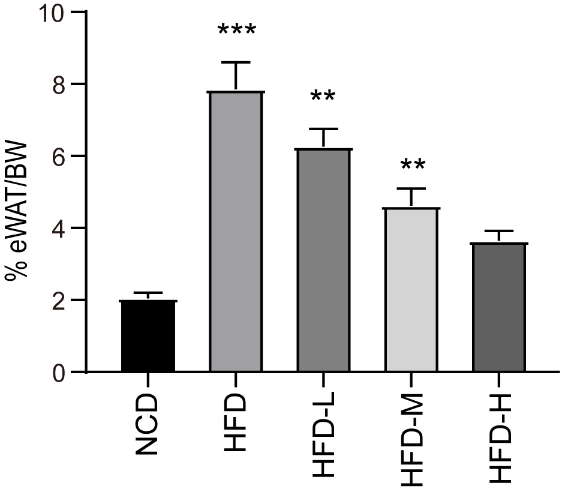
**

**Supplementary Table 1: The primer sequences used for qPCR**

| Gene | Primers |
| --- | --- |
| Srebp-1c | F: CTTACCCCTCCACCCTCAGA |
|  | R: TGTCGGGCTCAGAGTCACTA |
| Fasn | F: CTCCGAGGAACGAACACTGG |
|  | R: AGGCATTCTGTAGTGCCAGC |
| Scd-1 | F: GGCTTCCACAACTACCACCA |
|  | R: GTGACTCCCGTCTCCAGTTC |
| PPARa | F: TTCCAGCCCTTCCTCAGTCA |
|  | R: GCTCCGATCACACTTGTCGT |
| Cpt-1a | F: ATCGGACCCTAGACACCACT |
|  | R: AGTCATGATGATCGCCACCC |
| Acox1 | F: TGGTAGTCCGGAGAACACCC |
|  | R: TAACGCTGGCTTCGAGTGAG |
| Mcad | F: CATTCCGGAAAGTTGCGGTG |
|  | R: CACCCCTGTACACCCATACG |
| Pepck | F: GCTGGATGTCGGAAGAGGAC |
|  | R: CACCACATAGGGCGAGTCTG |
| G6pase | F: TGTCCCGGATCTACCTTGCT |
|  | R: AGAATCCAAGCGCGAAACCA |
